# The future of the Amazon: new perspectives from climate, ecosystem and social sciences

**DOI:** 10.1098/rstb.2008.0011

**Published:** 2008-02-11

**Authors:** Richard A Betts, Yadvinder Malhi, J. Timmons Roberts

**Affiliations:** 1 Met Office Hadley CentreExeter EX1 3PB, UK; 2 Environmental Change Institute, Oxford University Centre for the EnvironmentSouth Parks Road, Oxford OX1 3QY, UK; 3 The College of William and MaryWilliamsburg, VA 23187, USA

**Keywords:** tropical forest, deforestation, climate change, fire, Amazonia, Brazil

## Abstract

The potential loss or large-scale degradation of the tropical rainforests has become one of the iconic images of the impacts of twenty-first century environmental change and may be one of our century's most profound legacies. In the Amazon region, the direct threat of deforestation and degradation is now strongly intertwined with an indirect challenge we are just beginning to understand: the possibility of substantial regional drought driven by global climate change. The Amazon region hosts more than half of the world's remaining tropical forests, and some parts have among the greatest concentrations of biodiversity found anywhere on Earth. Overall, the region is estimated to host about a quarter of all global biodiversity. It acts as one of the major ‘flywheels’ of global climate, transpiring water and generating clouds, affecting atmospheric circulation across continents and hemispheres, and storing substantial reserves of biomass and soil carbon. Hence, the ongoing degradation of Amazonia is a threat to local climate stability and a contributor to the global atmospheric climate change crisis. Conversely, the stabilization of Amazonian deforestation and degradation would be an opportunity for local adaptation to climate change, as well as a potential global contributor towards mitigation of climate change. However, addressing deforestation in the Amazon raises substantial challenges in policy, governance, sustainability and economic science. This paper introduces a theme issue dedicated to a multidisciplinary analysis of these challenges.

## 1. Introduction

This theme issue arises from an international workshop held at Oriel College, Oxford, UK, in March 2007, and brings together 25 original pieces on some of the latest research about the drivers, processes and impacts of environmental changes in the Amazon and some potential new policy responses. It addresses a number of crucial questions regarding the current environmental change taking place. How much deforestation has occurred, what is driving it, how fast is it taking place now and how much do we expect in the future? What are the mechanisms linking global climate change to the Amazon-specific climate change, and how well are they understood? Is the climate of the region changing, and how do deforestation and climate change interact? Will anthropogenic global warming really lead to severe drought and forest dieback in the Amazon, as famously predicted by the Met Office Hadley Centre model (Betts *et al*. 2004; Cox *et al*. 2004)? How well do we understand the resilience or vulnerability of the Amazonian forests to drought, fires and other aspects of climate change? Finally, how are humans responding: what environmental policies are likely to be effective in preserving the forest while improving the welfare of the inhabitants of the region?

## 2. Human drivers of land cover change

The most visible and imminent threat to the Amazon forest is direct deforestation, accompanied by the degradation of surrounding forests by logging and the accidental spreading of fire from agriculture. By 2001, approximately 837 000 km^2^ of the Amazonian forests had been cleared, approximately 13% of their original extent (6.2 million km^2^), with gross rates over the 1990s being 25 000 km^2^ yr^−1^. Approximately 80% of this deforestation has been in Brazil, where it has been driven by planned expansion of roads into forest frontier regions, accompanied by unofficial unplanned roads, by cattle ranching and, more recently, soya bean agro-industry. In recent years, there has been a substantial reduction in Brazilian deforestation rates from a peak of 27 400 km^2^ yr^−1^ in 2004 to approximately 11 000 km^2^ yr^−1^ in 2007, owing to falling prices for soya and active Brazilian government intervention.

Perz *et al*. (2008) examine the dynamics surrounding planned and unplanned road expansion and land-use change. Nepstad *et al*. (2008) provide an overview of the dynamics where the intensive use of fire in preparing land for agriculture and the leakage of this fire frequently degrades any surrounding remaining forests, potentially pushing the whole region through an eco-climatic ‘tipping point’ into a more degraded scrub system. Regional and global economic forces drive local actors to take these actions. A new challenge arises from the potential expansion of biofuels or agroenergy: Sawyer (2008) and Nepstad *et al*. (2008) put this threat into context and explore its implications for future deforestation. The history of the Amazon region has been one of the booms and busts, as new valuable products are discovered, exploited and then replaced by cheaper alternatives. This history has been told many times, but Sawyer's piece uses that history to look into the Amazon's future. With much uncertainty and controversy surrounding biofuels as sustainable alternatives to fossil fuels, the current biofuel boom could be quite rapidly replaced by a bust. That is, the current wave of destruction in the Amazon could leave lasting consequences for the natural ecosystem but few lasting social benefits.

## 3. Climate/atmospheric change and vulnerabilities of the forest to drought

While deforestation is the most visible threat to the Amazon ecosystem, climate change is emerging as a creeping threat to the future of the region. Global atmospheric change has emerged as one of the greatest challenges of our century. In the context of the Amazon, the major agent of change in the forest ecosystem would most likely be decreased dry-season precipitation. Of the 23 global climate models employed by the Intergovernmental Panel on Climate Change (IPCC) in their 2007 report, 50–70% predict a substantial (above 20%) reduction of dry-season rainfall in eastern Amazonia under mid-range greenhouse gas emissions scenarios, 40% in central Amazonia and 20% in the west (Malhi *et al*. 2008). Water stress may be compounded by rising air temperatures, which in Amazonia have been rising at a rate of 0.25°C per decade (Malhi & Wright 2004), and are projected to rise by 3–8°C over the twenty-first century.

With the ecological and human systems of the Amazon being generally adapted to high rainfall and an infrequent fire regime, the occurrence of drought can provide a key driver of variability and change in both forested and agricultural areas. This volume presents results from a number of studies exploring the response of Amazonian systems to fire and drought.

The responses of intact fire-free forest to drought have recently been examined in two pioneering and ambitious experiments, in which a fraction of the precipitation falling through one hectare of rainforest canopy was collected and prevented from entering the soil over several years (Brando *et al*. 2008; Meir *et al*. 2008). The forests seem fairly resilient to a short-term drought, but persistent drought begins to cause a breakdown of forest structure. After 3 years of drought, death of large trees begins to occur; although these large trees may have deep roots and can access deep soil moisture when the soil near the surface is dry, they are also exposed above the canopy and so are subject to intense radiation stress (Brando *et al*. 2008). In a similar drought experiment, the drying of the soil was found to lead to a decrease in carbon release by respiration and hence an enhanced carbon sink in the short term (Meir *et al*. 2008), although this would turn into a carbon source in the face of long-term degradation.

Hence the response of forests to persistent drought has intrinsic inertia and has the potential to be slow and gradual, but these dynamics are fundamentally altered when fire is brought into the equation. Aragão *et al*. (2008) present a satellite remote-sensing analysis of the relationship between drought and fire incidence and argue that the leakage of fires from agricultural areas into surrounding forests is the most pervasive and threatening synergy between deforestation and climate change. There have been very few studies of the impacts of fires on the Amazon forest biodiversity, biomass and function: Barlow & Peres (2008) present detailed results from a path-breaking study in eastern Amazonia. Their study fills in the details of a harrowing picture of one possible future for Amazonia: a forest split into small fragments, degrading through fire leakage from surrounding agropastoral areas, drying through both global climate change and local deforestation, a degraded biodiversity-poor, low biomass scrub where the world's most biologically rich ecosystem once stood.

Some areas of forest may be more vulnerable than others to changes in the large-scale climate associated with drought. For example, the northwest of Amazonia currently has high rainfall caused by trade winds squeezing against the Andes mountains, and so may be less close to critical thresholds of precipitation. Also, some smaller areas of the southwest Amazonia adjacent to the Andes receive high rainfall due to orographical effects. These areas may act as refugia under scenarios of climate variability or change; a key conservation priority is to preserve these potential refugia and maintain their connectivity to the wider forest landscape (Killeen & Solórzano 2008).

## 4. Recent drought, its impacts and human responses

The 2005 drought in Amazonia raised awareness of the potential threat of a more drought-prone Amazon, and also provided an opportunity to study the responses of the human and ecological system to such drought events. Marengo *et al*. (2008) explore the climatology of this event, and Aragão *et al*. (2008) explore its impacts on fire occurrence. While previous drought events had been linked to anomalously warm sea surface temperatures (SSTs) in the equatorial Pacific associated with El Niño, Marengo *et al*. (2008) show that the reduced precipitation in 2005 was linked to a warm anomaly in the tropical North Atlantic.

A key question is whether there is a general trend towards drought conditions and, if so, whether this is associated with anthropogenic climate change. Li *et al*. (2008) show that the standard precipitation index (SPI), a measure of changes in precipitation normalized by the standard deviation, does indeed suggest a more pervasive drying trend over the southern Amazon. Using the international set of climate simulations assessed in the recent IPCC Fourth Assessment Report as a guide, the trend in SPI cannot be explained by natural internal variability of the climate system. However, it is not yet possible to attribute the observed changes to anthropogenic climate change.

One notable political response to the 2005 drought was the establishment of a ‘situation room’ by the state government of Acre, Brazil (the region at the epicentre of the drought), where information from climate models, near real-time satellite image analysis of drought and fires, and other sources was gathered and assessed in order to coordinate and focus state and national efforts (Boyd 2008). Based on this information, daily email briefings went to inform the deployment of local authorities and Brazilian army units fighting fires; still in many cases, the fires were too overwhelming to be brought under control. What remains today, however, is a networked set of actors and institutions which is better at collecting detailed and timely environmental information and in acting upon that information: this social system has become somewhat more ‘adaptive’. Such a system may be an early model of the adaptation response required by Amazonian governments in the face of increasing drought frequency.

In agricultural areas, the ability of farmers to adapt to climate variability and change is a crucial aspect of their vulnerability. Brondizio & Moran (2008) found that over 50% of farmers in their household surveys did not remember the gravity of one of the strongest droughts on record which occurred just 4 years earlier. They found that climate data were provided at the wrong scale (at the state or regional level, without real local information), and that there was a serious lack of agricultural extension assistance and community organization input into information provision. A major hurdle to adaptation by farmers was the extremely high turnover rate of people responsible for farms: newcomers lacked nuanced understandings of the local climate and ecosystem and also social networks that would provide them with better information and support during droughts. Crucially, communities of newcomers were less likely to be able to organize the cooperation needed to prevent fires from spreading accidentally.

## 5. Regional climate change due to future global warming

Previously, much attention has been given to the results of the Met Office Hadley Centre climate model, which suggests a strong drying of the Amazonian climate over the twenty-first century, resulting in a so-called ‘dieback’ of the forest. In the most extreme simulation, produced by a version of the model which includes feedbacks from the forest dieback and global soil respiration on global and regional climate change under medium–high emission scenarios, the annual precipitation averaged across Amazonia is reduced from approximately 1800 mm at the present day to less than 1100 mm by the 2040s, levels that are more typical of semi-arid savannah regions. By the 2070s, the simulated precipitation falls to less than 800 mm. While the magnitude of this reduction depends on the feedbacks from the forest itself on the regional water cycle, the Hadley Centre model still simulates some drying of the region even if the forest cover is maintained at the present-day state (Betts *et al*. 2008).

One of the key advances presented in this volume is a new understanding of distinct roles played by anomalous warming of the tropical Atlantic and Pacific sea surface in affecting rainfall in Amazonian dry seasons and wet seasons, respectively (Good *et al*. 2008; Harris *et al*. 2008). These regions affect the Hadley–Walker atmospheric circulation that in turn causes shifts in the location and magnitude of convective rainfall. Precipitation in the dry season over much of Amazonia is reduced as a result of changes in North Atlantic SSTs, and although the SST changes in this region would by themselves lead to an increase in wet-season rainfall, this is more than offset by the influence of tropical Pacific SSTs' positive anomalies that act to decrease wet-season rainfall (Harris *et al*. 2008). The critical threshold in ecosystem viability is reached when there is insufficient wet-season rainfall to fully recharge soil water reserves depleted in the preceding dry season.

Huntingford *et al*. (2008) find that the simulated drying and forest dieback in Amazonia in the Hadley Centre model is robust to changes in key parameter settings in the model, with different settings giving different degrees of drying and dieback, but all combinations of settings predicting drying and dieback to some degree. Looking at all climate models used by the IPCC Fourth Assessment Report, there is no consistent trend predicted in annual precipitation, but most models tend to demonstrate a reduction in dry-season rainfall, particularly in eastern Amazonia (Malhi *et al*. 2008). Li *et al*. (2008) take this analysis one step further. Rather than treating all climate models as equally valid, they attach greater weighting to those that are more able to reproduce the observed drying trend in recent decades. Once this is done, the estimated probability of drying of Amazonia over the twenty-first century is increased over that calculated from simple averaging.

## 6. Ecosystem responses to climate change

In the Hadley Centre coupled climate–vegetation model, the warming, drying climate reduced the mean net primary productivity, NPP, across Amazonia by approximately 52% by 2050 under a medium–high greenhouse gas emissions scenario (Harris *et al*. 2008). When the direct effects of CO_2_ on plant physiology are included in the model, NPP still reduces but to a lesser extent of 33% (Harris *et al*. 2008) due to the enhancement of photosynthesis by CO_2_ fertilization (Lloyd & Farquhar 2008). These responses are qualitatively robust to changes in the model formulation such as introduction of an improved response of photosynthesis to solar radiation, and an improved representation of vegetation dynamics (Huntingford *et al*. 2008).

When the components of the climate-only influence on NPP are isolated, the changes in SST patterns alone cause a 30% reduction in NPP mainly because of the shifts in atmospheric circulation and precipitation reduction (Harris *et al*. 2008). The local warming associated with global warming alone (with no changes in SST pattern) is simulated to lead to a 23% reduction in NPP. This suggests that while the changes in regional precipitation provide the dominant impact on Amazonian ecosystems in these climate scenarios, there would still be an impact due to warming alone. The reductions in NPP simulated under a long-term drying scenario are similar to those observed over 3–5 years in drought experiments (Brando *et al*. 2008). However, Lloyd & Farquhar (2008) argue from consideration of plant physiology that the acclimation of plant photosynthesis to higher temperatures is not considered in most vegetation models. Coupled with the decreased transpiration, increased plant water use efficiency and direct CO_2_ fertilization of photosynthesis, there may be a sustained increase in NPP that, in the absence of substantial drought, would persist well into the twenty-first century. Observational evidence of such increase in NPP and biomass is provided by long-term forest plots being monitored by the RAINFOR programme (Phillips *et al*. 2008). Their estimated carbon sink in intact, old-growth Amazonian rainforests is 0.6 Pg C yr^−1^, similar in magnitude to the carbon source from Amazonian deforestation, and an additional ecosystem service provided by the Amazonian forest. These authors emphasize, however, the uncertainty as to how long this sink will persist in the face of climate change, particularly when ecological interactions are considered. One such possible ecological interaction is fast-growing species such as pioneer trees and lianas gaining an advantage from higher CO_2_ concentrations levels, increased forest turnover and gap formation (Phillips *et al*. 2008). With faster-growing species having lower wood density, higher turnover and hence storing less carbon than slower-growing species, a shift in the competitive balance could lead to a *reduction* in overall ecosystem carbon stocks and hence a net emission of CO_2_. Currently, no vegetation models are capable of adequately representing such ecological processes.

An alternative approach to assessing longer term responses of Amazonian ecosystems to climate change is via the palaeoecological record (Bush *et al*. 2008; Mayle & Power 2008). In the Early-Mid Holocene period (8000–4000 years BP), the southern Amazon was substantially drier than the present day because of precessional changes in the seasonality of solar irradiance. Yet there is little evidence of large-scale replacement of forest by savannah except at the margins (Mayle & Power 2008). The evidence suggests some inherent resilience to drier conditions, at least in the absence of widespread fire ignition by human activity (Bush *et al*. 2008).

The highly biodiverse montane regions (the Andes, and the Brazilian and Guyanan shields) warrant particular attention, both as potential refugia for warming intolerant lowland species (Killeen & Solórzano 2008) and as vulnerable ecosystems in their own right. Warming and drying of the climate will lead to an increase in the altitude of particular temperature zones (Bush *et al*. 2008) and other key meteorological quantities such as the lifting condensation level (Cowling *et al*. 2008). Species and indeed whole ecosystems may therefore need to migrate upslope in order to remain within the conditions to which they are adapted, as they did at the last glacial–interglacial transition (Mayle & Power 2008). The rate of warming at the last transition was 10–100 times slower than that expected over the twenty-first century, however, and the ability of species to migrate at sufficient rates remains an unexplored question.

## 7. Interactions between climate change and direct human effects

With both land-use and climate changes being potential drivers of change in Amazonia, it is important to consider how these two drivers will interact. Bush *et al*. (2008) use charcoal records to study the fire history of the region over last 2000 years, and find links to both natural cycles and changes in human society. Over most of the past two millennia, peaks in fire activity coincide with peaks in solar forcing, suggesting variability in fire regimes driven by naturally forced climate variability. However, fire peaks decline after the indigenous population crash *ca* 1700 caused by European conquest and disease advance, suggesting an anthropogenic component to the fire regime in addition to the natural component. The lesson from prehistory is that climatic change makes the forests potentially vulnerable, but humans are needed to light the fires that can lead to retreat of forest.

A reduction in the Amazon forest cover, whether caused directly by deforestation or indirectly by climate change, could lead to further effects on climate at both local and global scales (Betts *et al*. 2008). Reduction in forest cover leads to changes in evaporation and the surface energy balance, which further reduces precipitation, especially further inland. Increases in the lifting condensation level due to climate change may be enhanced further by loss of the forest and reductions in evaporation (Cowling *et al*. 2008). Moreover, changes in carbon uptake and release affect the rate of increase of CO_2_ in the atmosphere, both as a consequence of direct emissions from forest loss and as a result of a lost opportunity for uptake of carbon in response to CO_2_ rise. The loss of substantial areas of Amazonian forest, from whatever cause, would therefore contribute to an acceleration of the rate of global climate change which again magnifies the regional drying in Amazonia (Betts *et al*. 2008).

Changes in global vegetation cover in the Hadley Centre model may also modify the climate through other geochemical cycles, specifically the emissions of mineral dust and isoprene (Betts *et al*. 2008). These processes have not yet been included in current Earth System Model simulations, but have the potential to exert further impacts on the climate system.

## 8. Climate and conservation policies and the Amazon forest

Following the 2007 United Nations Climate Conference in Bali, the negotiations on an international framework for reducing greenhouse gas emissions now also include a component known as Reducing Emissions from Deforestation and Degradation (REDD).^1^ The exact mechanisms are still to be negotiated, but the general principle would be that countries or stakeholders that own forests, and who would otherwise have continued with deforestation, would receive payments for reducing further deforestation. Several papers in this volume discuss both the potential and the implementation of challenges that REDD faces as an effective mechanism to maintain the extent and resilience of the Amazon forest and its human and non-human denizens.

Ebeling & Yasué (2008) discuss how the inclusion of REDD credits in international carbon markets could generate billions of euros of carbon finance with as little as 10% reduction in deforestation. REDD could also provide co-benefits for biodiversity conservation and social justice; however, there are still significant challenges to its effectiveness (Ebeling & Yasué 2008; Hall 2008). There is a need to reward only genuine reductions in deforestation and to avoid penalizing countries which already have achieved lowered rates of deforestation. There are risks of ‘leakage’ of deforestation, i.e. displacement of the deforestation activity to another location, but Ebeling & Yasué suggest that this risk could be reduced by awarding REDD credits at the national level rather than at the project level.

Permanence of forest conserved under REDD is also an issue, especially given the possibility of detrimental climate changes that may lead to dieback of forests that have been saved from deforestation (Ebeling & Yasué 2008). It might be argued that such a situation would result in an opportunity cost of avoiding deforestation (e.g. preventing the land from being turned over to mining or agricultural production), but without the benefit of mitigating climate change. Ebeling & Yasué make the case that this argument is too simplistic, as it ignores the benefits of delaying emissions even if emissions still ultimately occur; the cumulative accumulation of gases and warming effect would still have been reduced. Furthermore, we have argued above that severe forest loss due to climate change is a possibility but not highly probable in the absence of further expansion of direct deforestation. Indeed, REDD would not only be a direct *mitigation* strategy reducing CO_2_ emissions, but also a regional *adaptation* strategy reducing the risk of fire-driven dieback of remaining Amazonian forest and maintaining the eco-climatic resilience of the biome.

REDD has the potential to generate greater financial resources for forest protection, but this needs to be coupled with thoughtful and forward-thinking management of reserves and the land between them; this may help to minimize the impacts of climate change on biodiversity (Sawyer 2008). An example of the kind of thinking required is provided by Killeen & Solórzano (2008) in the context of migration corridors to thermal refugia on the slopes of the Andes. An unanticipated migration challenge in this region is the presence of the low Andean front range, largely separated from the main Andean range by lowland forests. Under warming conditions, it is possible that species may migrate onto the low front range, but without intervention would then be trapped and unable to traverse the lowland forest gap to reach the main Andean range. However, Killeen & Solórzano identify a few critical regions where there is a connectivity between the front and main ranges; these regions may act as effective conduits for species migration and are clear conservation priorities. Such strategies for ‘weathering the storm’ could be a critical adaptation strategy carried out in synergy with mitigation efforts such as REDD, and may be especially important if there is a need to deal with an ‘overshoot’ scenario in which climate change exceeds an intended target and is then subsequently reduced (IPCC 2007). However, Nepstad *et al*. (2008) point out that simply conserving forest cover by eliminating direct deforestation may not be enough: protection of the forest may also need active fire control when droughts occur, as was pioneered in Acre in 2005 (Boyd 2008).

The funds generated through REDD in themselves will not automatically slow down deforestation; the effective implementation of REDD will confront many issues, including ethical concerns, local land-use rights and social justice, effective monitoring capacity and poor governance of the forest frontier (Hall 2008; Lemos & Roberts 2008). Poor governance can create a barrier between the receipt of international funding and the actual implementation of efforts for reducing deforestation. In their global assessment, Ebeling & Yasué (2008) find that those countries with the greatest opportunity for financial benefit from REDD credits may not have the appropriate governance structures to actually implement reduced deforestation policies effectively. Hall (2008) presents an important case study of a Brazilian scheme that attempted to provide payment for ecosystem services to populations using the forest sustainably, but which has not (as yet) provided the promised payments to small landowners. Co-benefits of REDD for social justice may be difficult to achieve, as the most efficient means of implementing REDD policies would be to target the small number of major landowners rather than the very numerous smaller landowners who would have the greatest need for the financial support (Hall 2008). Hall's piece provides a cautiously optimistic endorsement of why REDD is needed and some initial lessons on how it would have to be administered to be effective.

REDD policies are likely to require a strong network of support in order to be effective, including a wide range of stakeholders such as indigenous communities, industry, and regional and national governments (Lemos & Roberts 2008). This requires an engagement with these groups rather than a focus simply on ecosystems themselves. The World Bank, for example, shifted in the 1990s to recognize that protection of the Amazon ecosystems required support and outreach to broad segments of society and not just procedures to manage ecosystems. This is obviously difficult, and in the case of potentially paying for reduced deforestation, large portions of local economies may be eliminated, such as those involved in extracting, processing, transporting and marketing local products. Lemos & Roberts argue that an alternative for local economies will require much more than just payments that cover ‘opportunity costs’ narrowly defined—many powerful actors will lose out in such scenarios and may resist the programme's implementation.

Perz *et al*. (2008) describe how most proposals for addressing uncontrolled deforestation are either ‘state based’ or ‘community based’. State-based approaches call for regulations, enforcement, the creation of parks and reserves, and fiscal incentives for more sustainable management of lands. On the other hand, community-based approaches—established by indigenous groups and longstanding residents like rubber tappers and riverine peoples—manage natural resources from ‘the bottom-up’, by restricting access and creating local rules about overuse and systems of enforcing compliance. Under extreme pressures from global economic and national forces, however, Perz *et al*. (2008) argue that a ‘hybrid approach’ is needed, which would have the state providing the resources and oversight for largely local consultation and development of resource management. They examine the three-nation MAP initiative (between Brazil, Peru and Bolivia) as one of the region's first efforts at ‘hybrid governance’ by combining infrastructure improvement and economic development in the region with some efforts to protect ecosystems.

The subject of mitigating and adapting to climate change is probably the most dynamic current interface between natural science, social science and economics, and regional, national and international policy. As such, it raises challenges in creating effective dialogue between these arenas. Alves (2008) discusses the process of science influencing environmental policy, and notes that it is not enough to focus purely on the natural sciences. Even if the natural science studies are multidisciplinary and integrated in their investigations of processes of change in regions such as the Amazon, this fails to recognize that humans are also fully part of the system. Proper inclusion of the social sciences into multidisciplinary studies is crucial if the results are to be relevant to policy. Much of the work on changes in climate, ecology and land use in Amazonia over the past decade has taken place through the large-scale atmosphere–biosphere experiment in Amazonia (LBA) that has its roots in climatological studies. Social science perspectives have begun to be brought into LBA and are increasing in prominence; Alves (2008) warns that badly flawed policies have been deployed in the past in the Amazon when social and political factors are not well understood.

## 9. Conclusions

This volume brings together 25 papers on some of the latest research on the drivers, processes and impacts of environmental changes in the Amazon and potential public policy responses. Despite a substantial slowdown in deforestation in Brazil over the past 3 years, the Amazon remains subject to an unquestionable threat from deforestation and degradation, arising mainly from ranching, farming, road building and logging. Past rebounds in deforestation rates have shown how conservation efforts can founder in the face of the twin pressures of a global economy offering higher prices for commodities and national governments seeking to accelerate their national economies by integrating the productive capacity of their Amazon regions. What is new and still uncertain is that there may also be a major compounding threat from global warming, particularly through a potential increase in drought risk. Recent drying trends in Amazonia show some similarities to those projected in computer models, but it is not yet possible to attribute these events to anthropogenic climate change. While the forest may be resilient to some level of drought or other climate change, the additional impacts of deforestation and degradation may reduce this resilience. Therefore, it is not a case of simply establishing whether deforestation or climate change is more important—the two threats may compound each other and hence should be considered together when planning and implementing land-use or conservation policies in Amazonia. The inclusion of carbon credits for REDD within the Kyoto Protocol has the potential to help slow deforestation to mitigate climate change and confer other benefits for biodiversity and human welfare; however, there are still a number of challenges to this. The challenge of maintaining the ecosystem services of Amazonia (and other regions) in the face of deforestation pressures and climate change will require interdisciplinary research and analyses that span the climatological, ecological, social, political and economic sciences, and interface effectively with regional and international policy. We hope that this volume makes a useful contribution to this effort.

## Endnote

1 There is some confusion in usage of the acronym REDD. It is most commonly used as an abbreviation for Reduced Emissions from Deforestation and Degradation. Official UNFCCC documentation tends to refer to Reduced Emissions from Deforestation in developing countries, but abbreviates this to RED-dc, not REDD. The acronym RED is also used when only deforestation is discussed and not degradation, as in some papers in this volume.
